# A Proof of Concept of the Role of TDM-Based Clinical Pharmacological Advices in Optimizing Antimicrobial Therapy on Real-Time in Different Paediatric Settings

**DOI:** 10.3389/fphar.2021.755075

**Published:** 2021-09-27

**Authors:** Milo Gatti, Pier Giorgio Cojutti, Caterina Campoli, Fabio Caramelli, Luigi Tommaso Corvaglia, Marcello Lanari, Andrea Pession, Stefania Ramirez, Pierluigi Viale, Federico Pea

**Affiliations:** ^1^ Department of Medical and Surgical Sciences, Alma Mater Studiorum University of Bologna, Bologna, Italy; ^2^ SSD Clinical Pharmacology, IRCCS Azienda Ospedaliero-Universitaria di Bologna, Bologna, Italy; ^3^ Infectious Diseases Unit, IRCCS Azienda Ospedaliero-Universitaria di Bologna, Bologna, Italy; ^4^ Pediatric Intensive Care Unit, IRCCS Azienda Ospedaliero-Universitaria di Bologna, Bologna, Italy; ^5^ Neonatal Intensive Care Unit, IRCCS Azienda Ospedaliero-Universitaria di Bologna, Bologna, Italy; ^6^ Pediatric Emergency Unit, IRCCS Azienda Ospedaliero-Universitaria di Bologna, Bologna, Italy; ^7^ Pediatric Oncology & Hematology Unit ‘Lalla Seràgnoli', IRCCS Azienda Ospedaliero-Universitaria di Bologna, Bologna, Italy; ^8^ LUM Metropolitan Laboratory, AUSL Bologna, Bologna, Italy

**Keywords:** clinical pharmacology advice, personalized antimicrobial therapy, neonatology, paediatric intensive care unit, paediatric emergency, paediatric onco-haematology

## Abstract

**Introduction:** Antimicrobial treatment is quite common among hospitalized children. The dynamic age-associated physiological variations coupled with the pathophysiological alterations caused by underlying illness and potential drug-drug interactions makes the implementation of appropriate antimicrobial dosing extremely challenging among paediatrics. Therapeutic drug monitoring (TDM) may represent a valuable tool for assisting clinicians in optimizing antimicrobial exposure. Clinical pharmacological advice (CPA) is an approach based on the correct interpretation of the TDM result by the MD Clinical Pharmacologist in relation to specific underlying conditions, namely the antimicrobial susceptibility of the clinical isolate, the site of infection, the pathophysiological characteristics of the patient and/or the drug-drug interactions of cotreatments. The aim of this study was to assess the role of TDM-based CPAs in providing useful recommendations for the real-time personalization of antimicrobial dosing regimens in various paediatric settings.

**Materials and methods:** Paediatric patients who were admitted to different settings of the IRCCS Azienda Ospedaliero-Universitaria of Bologna, Italy (paediatric intensive care unit [ICU], paediatric onco-haematology, neonatology, and emergency paediatric ward), between January 2021 and June 2021 and who received TDM-based CPAs on real-time for personalization of antimicrobial therapy were retrospectively assessed. Demographic and clinical features, CPAs delivered in relation to different settings and antimicrobials, and type of dosing adjustments were extracted. Two indicators of performance were identified. The number of dosing adjustments provided over the total number of delivered CPAs. The turnaround time (TAT) of CPAs according to a predefined scale (optimal, <12 h; quasi-optimal, between 12–24 h; acceptable, between 24–48 h; suboptimal, >48 h).

**Results:** Overall, 247 CPAs were delivered to 53 paediatric patients (mean 4.7 ± 3.7 CPAs/patient). Most were delivered to onco-haematological patients (39.6%) and to ICU patients (35.8%), and concerned mainly isavuconazole (19.0%) and voriconazole (17.8%). Overall, CPAs suggested dosing adjustments in 37.7% of cases (24.3% increases and 13.4% decreases). Median TAT was 7.5 h (IQR 6.1–8.8 h). Overall, CPAs TAT was optimal in 91.5% of cases, and suboptimal in only 0.8% of cases.

**Discussion:** Our study provides a proof of concept of the helpful role that TDM-based real-time CPAs may have in optimizing antimicrobial exposure in different challenging paediatric scenarios.

## Introduction

Severe bacterial infections are a growing problem in the pediatric population and the rise of multidrug-resistant (MDR) pathogens may seriously challenge optimal treatment (Hsu and Tamma, 2014). Antimicrobial use is quite common among hospitalized children, possibly exceeding 50% in different paediatric settings, but unfortunately most antimicrobials do not have specific paediatric posology based on pharmacokinetic and/or pharmacokinetic/pharmacodynamic studies that were carried out in this patient population (Korth-Bradley, 2018). Although the paradigm shift that states “children are not small adults” is widely accepted nowadays (Moore, 1998), in most cases dose scaling in pediatrics is still based on allometric scaling (Le and Bradley, 2018). This approach selects drug dosage on the basis of the non-proportional relationship that exists between the pharmacokinetic (PK) parameters, such as drug clearance and/or volume of distribution, and the body size descriptors, such as body surface area and/or lean body weight (Le and Bradley, 2018). Unfortunately, allometric scaling is far from being optimal, as it has some intrinsic limitations. Importantly, it does not take into account the process of organ maturation that occurs in the first years of life. It should not be overlooked that drug disposition in newborns, infants and toddlers may be affected by age-related factors both concerning organ development and maturation, and body composition, thus potentially rendering drug exposure unpredictable (Kearns et al., 2003). Renal function normalized to body weight (in terms of creatinine clearance in mL/min/kg) may be 2-3 fold higher during the first year of life than in adults (Hayton, 2000), and in the subsequent years progressively decreases reaching values similar to those of adults within 10 years of age (Funk et al., 2012). Additionally, allometric scaling does not consider the influence that some pathophysiological conditions may have in altering the pharmacokinetic behavior of some drugs. Interindividual pharmacokinetic variability, namely a well-known issue that may affect drug exposure and treatment outcomes (Collins and Varmus, 2015), may be especially relevant in the case of the critically ill and/or of the onco-hematological pediatric patients. The underlying presence of sepsis and/or septic shock, and/or of hematological malignancies like acute myeloid leukemia and/or acute lymphoblastic leukemia may frequently lead to the so-called augmented renal clearance, namely a pathophysiological condition that may significantly increase the renal clearance of hydrophilic drugs, as for example beta-lactams and/or aminoglycosides. Last, but not least, drug-drug interactions may furtherly make the implementation of appropriate antimicrobial dosing extremely challenging, as in the case of antifungal triazoles.

Therapeutic drug monitoring (TDM) may represent a valuable tool for assisting clinicians in optimizing antimicrobial dosing. However, for providing clinicians with optimal TDM-based dosing adjustments in each single patient, it is necessary that the results could be provided on real-time and that they are interpreted correctly. The clinical pharmacological advice (CPA) is an advice for optimizing drug exposure in each single patient that is delivered by the MD Clinical Pharmacologist who interprets on real-time the TDM results of antimicrobials in relation to some specific underlying conditions, namely the antimicrobial susceptibility of the clinical isolate, the site of infection, the pathophysiological characteristics of the patient and/or the potential drug-drug interactions of co-treatments.

The aim of this study was to provide a proof of concept of the role that TDM-based CPAs may have for real-time personalization of antimicrobial exposure in different paediatric settings.

## Materials and Methods

### Study Design

This is a proof-of-concept study that has the purposes of describing the organizational procedures of a newly established Clinical Pharmacology Unit focused at providing real-time CPAs for individualizing antimicrobial exposure in different specific paediatric settings, and of assessing the clinical impact of the CPAs in the first 6 months of activity.

### Organizational Procedures of the Clinical Pharmacology Unit

The IRCCS Azienda Ospedaliero-Universitaria of Bologna, Italy is a 1362-bed tertiary care teaching hospital which is currently organized in nine integrated activity Departments including 87 different operating units. The Clinical Pharmacology Unit was activated on November 2020, and since December 2020 started in providing educational webinars concerning the role of CPAs for real-time personalization of antimicrobial exposure that are based on TDM samples that are analyzed at the Unique Metropolitan Laboratory (LUM).

The CPA is an approach based on the correct interpretation of the TDM result by the MD Clinical Pharmacologist in relation to specific underlying conditions, namely the antimicrobial susceptibility of the clinical isolate according to the MIC provided by the Clinical Microbiologist, the site of infection, the pathophysiological characteristics of the patient (e.g., body mass index, renal function, sepsis, requirement for continuous renal replacement therapy or intermittent haemodialysis) and/or the influence of concomitant therapies. Clinical features of each patient (i.e., weight, height, diagnosis, concomitant therapies, date of starting antimicrobial therapy, drug dose and frequency of administration, time of blood sample collection) were supplied by the physician who requested the CPA and had the patient in charge. This approach allowed to provide dosing adjustment recommendations useful at optimizing drug exposure in each single patient.

The CPAs were provided five times weekly (from Monday to Friday) for 18 different antimicrobials: 13 antibiotics (piperacillin-tazobactam, ampicillin, meropenem, ceftazidime, cefepime, vancomycin, teicoplanin, amikacin, gentamicin, linezolid, levofloxacin, ciprofloxacin, and rifampicin), four antifungals (fluconazole, voriconazole, posaconazole, and isavuconazole), and one antiviral (ganciclovir) as detailed in Table 1. Optimal pharmacodynamic targets both for maximizing clinical efficacy and for minimizing resistance occurrence were considered a plasma steady-state concentration 4-fold above the minimum inhibitory concentration (MIC) (Css/MIC >4) for time-dependent antimicrobials, and a peak concentration to MIC ratio (Cmax/MIC) ≥ 8–12 for concentration-dependent antimicrobials (Abdul-Aziz et al., 2020) (Table 1 **)**. Blood samples for first TDM assessment were routinely collected at 48- and 72-h after the start of the treatment for beta-lactams and for other agents (e.g., linezolid, azoles), respectively, in order to ensure that steady state concentrations have been achieved. Re-assessments were commonly performed at least after 48-h the implementation of dosing adjustments recommended in CPAs.

**TABLE 1:** Scheduled timing, expected PK/PD target, and TDM-guided dosage adjustments of antimicrobials for which clinical pharmacological advice is available five-times weekly.

5 times weekly			
Antimicrobial	**Timing to CPA**	**Expected target**	**Dosage adjustment**
**Piperacillin-Tazobactam**	4–6 h for TDM result^c^	C_min_ or C_ss_ > 4xMIC	Reduction
			50% if C_min_ or C_ss_ > 10xMIC
	CPA within 2–4 h^d^		Increase
			50% if C_min_ or C_ss_ < 2xMIC
**Meropenem**	4–6 h for TDM result^c^	C_min_ or C_ss_ > 4xMIC	Reduction
			50% if C_min_ or C_ss_ > 10xMIC
	CPA within 2–4 h^d^		Increase
			50% if C_min_ or C_ss_ < 2xMIC
**Ceftazidime**	4–6 h for TDM result^c^	C_min_ or C_ss_ > 4xMIC	Reduction
			50% if C_min_ or C_ss_ > 10xMIC
	CPA within 2–4 h^d^		Increase
			50% if C_min_ or C_ss_ < 2xMIC
**Ampicillin-Sulbactam** **^b^**	4–6 h for TDM result^c^	C_min_ or C_ss_ > 4xMIC	Reduction
			50% if C_min_ or C_ss_ > 10xMIC
	CPA within 2–4 h^d^		Increase
			50% if C_min_ or C_ss_ < 2xMIC
**Cefepime** **^b^**	4–6 h for TDM result^c^	C_min_ or C_ss_ > 4xMIC	Reduction
			50% if C_min_ or C_ss_ > 10xMIC
	CPA within 2–4 h^d^		Increase
			50% if C_min_ or C_ss_ < 2xMIC
**Linezolid**	4–6 h for TDM result^c^	C_min_ 2–8 mg/L	Reduction
			50% if C_min_ > 10 mg/L
	CPA within 2–4 h^d^		Increase
			25–50% if C_min_ < 2 mg/L
**Vancomycin** **^e^**	4–6 h for TDM result^c^	C_ss_ 18–20 mg/L	Prefer CI and calculate correct dosing in order to achieve an AUC/MIC >400 according to C_ss_ and MIC
	CPA within 2–4 h^d^		
**Teicoplanin** **^e^**	4–6 h for TDM result^c^	C_ss_ 20–30 mg/L	Reduction
			25–50% if C_min_ > 35 mg/L
	CPA within 2–4 h^d^		Increase
			50% if C_min_ < 10 mg/L
**Amikacin** **^e^**	4–6 h for TDM result^c^	C_max_ 8-10xMIC	Reduction
			every 36–48 h if C_min_ > 1.5 mg/L
	CPA within 2–4 h^d^	C_min_ < 1 mg/L	Increase
			25% if C_max_ < 8xMIC
**Gentamicin** **^e^**	4–6 h for TDM result^c^	C_max_ 8-10xMIC	Reduction
			every 36–48 h if C_min_ > 0.5 mg/L
	CPA within 2–4 h^d^	C_min_ < 1 mg/L	Increase
			25% if C_max_ < 8xMIC
**Rifampicin**	4–6 h for TDM result^c^	C_max_ 8–22 mg/L	Reduction
			25–50% if C_max_ > 22 mg/L
	CPA within 2–4 h^d^		Increase
			50% if C_max_ < 4 mg/L
**Levofloxacin**	4–6 h for TDM result^c^	C_max_ 10xMIC	Reduction
			every 36–48 h if C_min_ > 2 mg/L
	CPA within 2–4 h^d^	C_min_ < 2 mg/L	Increase
			25% if C_max_ < 10xMIC
**Ciprofloxacin**	4–6 h for TDM result^c^	C_max_ 10xMIC	Reduction
			25% if C_min_ > 2 mg/L
	CPA within 2–4 h^d^	C_min_ < 2 mg/L	Increase
			25% if C_max_ < 10xMIC
**Fluconazole**	4–6 h for TDM result^c^	C_min_ 10–20 mg/L	Reduction: 50% if C_min_ > 50 mg/L 25% if C_min_ 30–50 mg/L
	CPA within 2–4 h^d^		Increase: 25% if C_min_ < 10 mg/L
**Voriconazole**	4–6 h for TDM result^c^	C_min_ 1–3 mg/L	Reduction
			stop if C_min_ > 8–10 mg/L 25-50% if C_min_ 3.5–8 mg/L
	CPA within 2–4 h^d^		Increase
			every 6–8 h if C_min_ < 1 mg/L
**Posaconazole**	4–6 h for TDM result^c^	C_min_ 1–3 mg/L	Reduction
			25–50% if C_min_ > 4 mg/L
	CPA within 2–4 h^d^		Increase
			every 12 h if C_min_ < 1 mg/L
**Isavuconazole**	4–6 h for TDM result^c^	C_min_ 1–7 mg/L	Reduction
			25–50% if C_min_ >8 mg/L
	CPA within 2–4 h^d^		Increase
			25–50% if C_min_ < 1 mg/L
**Ganciclovir/valganciclovir**	4–6 h for TDM result^c^	C_min_ 0.7–2 mg/L	Reduction
			stop if C_min_ > 5 mg/L 25-50% if C_min_ 2–5 mg/L
	CPA within 2–4 h^d^		Increase
			every 6–8 h if C_min_ < 0.5 mg/L

a available from April 01, 2021.

b available from May 27, 2021.

c if sanding samples occurs within 2.00 pm; 24 h for samples sent after 2.00 pm.

d after TDM results communication.

e fully automated analytic procedure.

f experimental analytic methods.

AUC, area under concentration-time curve; C_max_, peak concentration; C_min_, trough concentration; C_ss_, steady-state concentration; CI, continuous infusion; CPA, clinical pharmacology advice; MIC, minimum inhibitory concentration; PK/PD, pharmacokinetic/pharmacodynamic; TDM, therapeutic drug monitoring.

The CPAs were provided usually within 4 h after that the TDM results were made available in the hospital intranet system by the LUM. Blood samples for TDM were processed by the LUM in the same day if they were delivered within 2.00 p.m., otherwise they were processed in the subsequent day.

The CPAs had been adopted by four specific paediatric settings, namely paediatric intensive care unit [ICU], paediatric onco-haematology/transplant unit, neonatology, and emergency paediatric ward. Antimicrobial treatment and relative dosage were initially selected on the basis of dedicated national and local guidelines with the support of the infectious disease consultant, and successively adjusted according to the TDM-based CPAs. The relationship between antimicrobial exposure and clinical outcome in terms of efficacy and safety was regularly assessed during the delivery of CPAs.

### Data Analysis of CPAs Provided in the First Six Months of Activity

Paediatric patients who were admitted to the four different specific paediatric settings of the IRCCS Azienda Ospedaliero-Universitaria of Bologna, Italy and who required TDM-based CPAs for real-time personalization of antimicrobials between January 2021 and June 2021 were retrospectively assessed. Demographic and clinical features (age, gender, diagnosis), and CPAs were retrieved for each patient.

Two indicators of performance were defined in order to assess the usefulness of the CPAs for real-time optimization of antimicrobial dosing in the paediatric settings. First, the proportion of dosing adjustments (increase or reduction) recommended in the CPAs and applied by the attending clinicians over the total number of delivered CPAs in relation to the different settings and antimicrobials. Second, the turnaround time (TAT) of the antimicrobial CPA, defined as the timeframe elapsing between the delivery of the TDM sample to the LUM and the publication in the hospital intranet of the definitive TDM-guided CPA. The TAT of the antimicrobial CPA was defined as optimal, if < 12 h; quasi-optimal, if between 12–24 h; acceptable, if between 24–48 h and suboptimal, if > 48 h. The scale was predefined according to the feasibility in promptly providing paediatricians a real-time CPA, so that the recommended dosing adjustments could be implemented as quickly as possible after the delivery of TDM sample to the laboratory.

Data were expressed as mean ± standard deviation (SD) or median and interquartile range (IQR) according to data distribution, while categorical variables were expressed as count and percentage.

The study was approved by the local Ethic Committee (No. 443/2021/Oss/AOUBo).

## Results

### Organizational Aspects of the Clinical Pharmacology Unit

**FIGURE 1:**
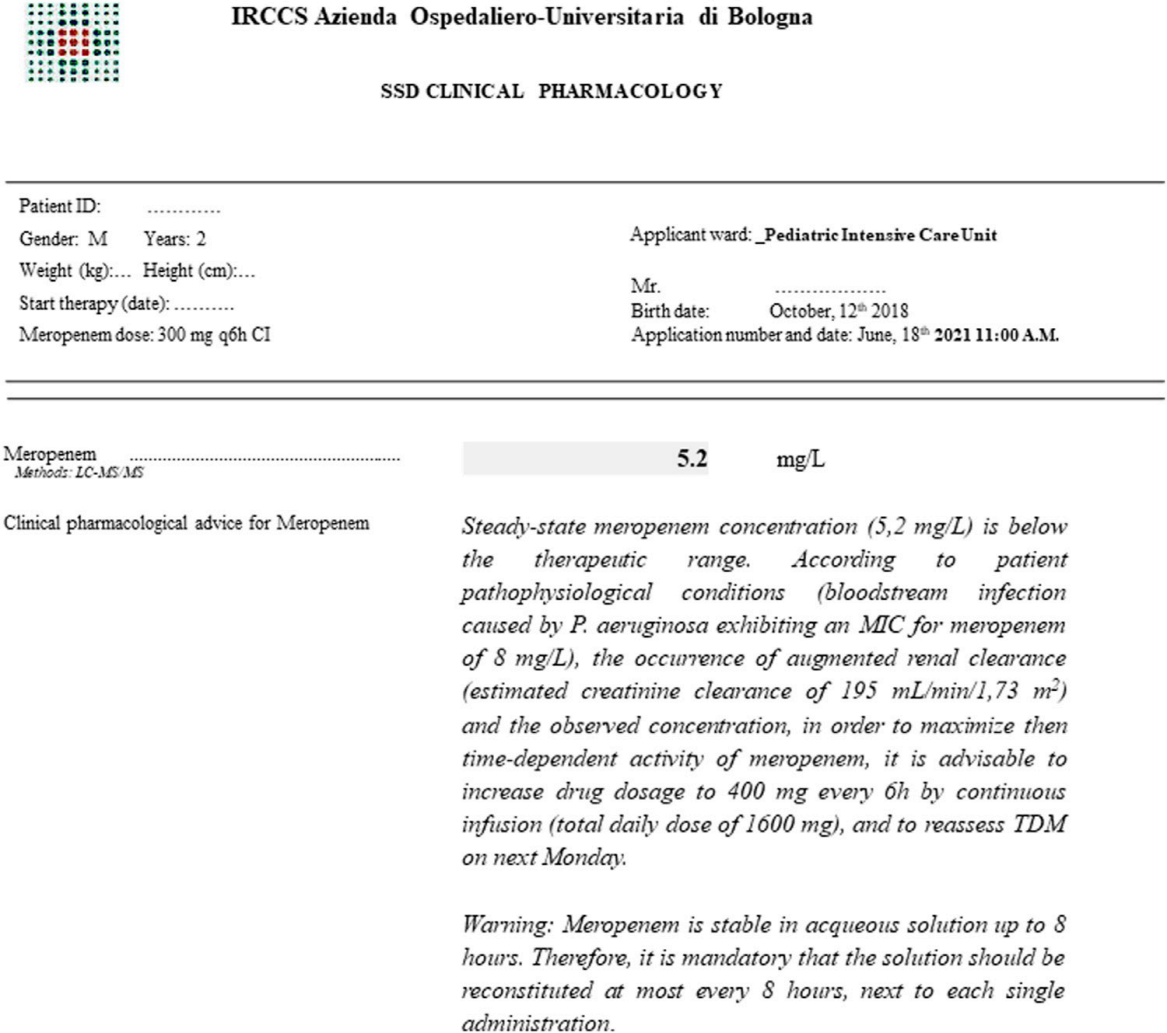
Example of a TDM-guided clinical pharmacological advice for personalizing antibiotic treatment with meropenem in a critically ill paediatric patient affected by augmented renal clearance (estimated creatinine clearance according to bedside revised Schwartz equation) and concomitant bloodstream infection due to *Pseudomonas aeruginosa* exhibiting a minimum inhibitory concentration for meropenem of 8 mg/L. SSD: departmental structure.

**FIGURE 2:**
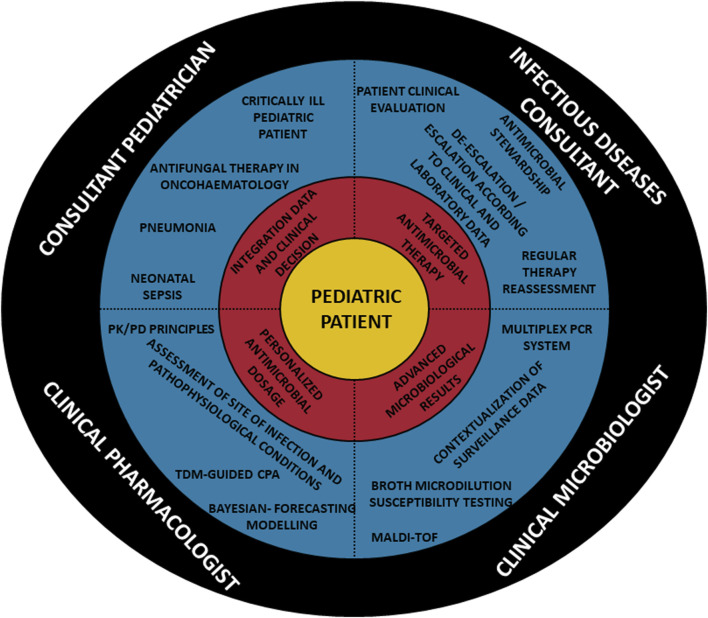
Features of multidisciplinary taskforce involved in the management of empirical and targeted antimicrobial therapy in paediatric patients. CPA, clinical pharmacological advice; MALDI-TOF, Matrix Assisted Laser Desorption Ionization–Time of Flight; PK/PD, pharmacokinetic/pharmacodynamic; TDM, therapeutic drug monitoring.

The Clinical Pharmacology Unit is included in the Department for the Integrated Management of Infective Risk of the IRCCS Azienda Ospedaliero-Universitaria of Bologna. The priority goal consists in providing personalized CPAs for both inpatients and outpatients. An example of CPA performed in a specific paediatric scenario is shown in Figure 1. In the early period (Jan-Mar 2021), the main activity was to provide educational interventions in order to increase the awareness of clinicians regarding the importance that a real-time and integrated personalization of antimicrobial therapy may have in challenging paediatric scenarios. These interventions were based on dedicated webinars and on a specific on field work at the bedside aimed to identify which paediatric patients could have benefit more by a personalized CPAs, and to stress the importance of correct timing for TDM blood sampling in relation to the time of drug administration. The individualization of antimicrobial therapy in the paediatric population was made possible thanks to the implementation of a multidisciplinary taskforce composed by the pediatrician, the infectious disease consultant, the clinical microbiologist and the MD clinical pharmacologist. The main features of members involved in the multidisciplinary team and their coordinated and synergistic activities is shown in Figure 2.

### Evaluation of the Impact of the CPAs in Different Hospital Paediatric Settings

Overall, during the 6-month period study, 247 CPAs were delivered to 53 paediatric patients (mean 4.7 ± 3.7 CPAs per patient). Median age of patients was 6 years (IQR 1–16 years), with no gender preponderance (male 49.1%). Leukemia and pneumonia were the most frequent underlying diseases, accounting for 39.6 and 20.8% of paediatric patients having CPAs, respectively (Table 2).

**TABLE 2:** Clinical and demographics features of paediatric patients in which at least one CPA for antimicrobial dosing adjustment was performed.

Demographics and clinical features	No. (%)
Age	
Median (years; IQR)	6 (1–16)
<1 year	9 (17.0%)
1–5 years	13 (24.5%)
5–12 years	15 (28.3%)
12–17 years	16 (30.2%)
Sex	
Male	26 (49.1%)
Female	27 (50.9%)
Weight	
Median (kg; IQR)	20 (11.1–31.8)
Height	
Median (cm; IQR)	120 (75.3–147)
Setting	
Neonatology	5 (9.4%)
Paediatric ICU	19 (35.9%)
Paediatric onco-haematology/Transplant Unit	21 (39.6%)
Emergency paediatric ward	8 (15.1%)
Underlying disease	
AML	12 (22.6%)
ALL	9 (17.0%)
HAP	9 (17.0%)
Solid neoplasms	4 (7.5%)
Meningo-ventriculitis	3 (5.7%)
Abdominal perforation	3 (5.7%)
Encephalopathy	3 (5.7%)
Neonatal sepsis	2 (3.8%)
CAP	2 (3.8%)
Necrotizing enterocolitis	2 (3.8%)
Others	4 (7.5%)

ALL, acute lymphatic leukaemia; AML, acute myeloid leukaemia; CAP, community-acquired pneumonia; HAP, hospital-acquired pneumonia; ICU, intensive care unit; IQR, interquartile range.

Most of the patients who received the CPAs were admitted to the paediatric onco-haematology/transplant unit (21; 39.6%) and to the paediatric ICU (19; 35.8%), and overall received more than 80% of the total CPAs. The mean (±SD) number of CPAs per patient was higher in the paediatric onco-haematology/transplant unit and in the emergency paediatric ward, and amounted respectively to 6.2 ± 4.2 and 4.4 ± 4.9 (Table 3).

**TABLE 3:** Number of clinical pharmacological advice performed in different paediatric settings.

Paediatric setting	No. of patients	Median age (IQR; years)	Male proportion	No. of CPA	CPA/patient
Neonatology	5	0.08 (0.06–0.16)	4 (80.0%)	7	1.4 ± 0.9
Paediatric ICU	19	4 (1.5–15)	5 (26.3%)	75	3.9 ± 2.1
Oncohaematology/Transplant Unit	21	9 (6–18)	13 (61.9%)	130	6.2 ± 4.2
Emergency paediatric ward	8	12.5 (5–18)	4 (50.0%)	35	4.4 ± 4.9
Overall	53	6 (1–16)	26 (49.1%)	247	4.7 ± 3.7

CPA, clinical pharmacology advice; ICU, intensive care unit, IQR: interquartile range

The CPAs concerned 17 out of the 18 antimicrobials for which the TDM was available, and were provided mainly for antibiotics (121; 49.0%) and for antifungals (112; 45.3%). Overall, 84.0% of the CPAs delivered to the paediatric ICU concerned antibiotics, and 78.5% of those delivered to the paediatric onco-haematology/transplant unit regarded azole antifungals. The total number of CPAs requested for each antimicrobial were ≥10 for four antibiotics (piperacillin-tazobactam, meropenem, linezolid, and teicoplanin), four antifungals (isavuconazole, voriconazole, posaconazole, and fluconazole), and for the antiviral ganciclovir (Table 4). The most frequently requested CPAs concerned isavuconazole (47; 19.0%) and voriconazole (44; 17.8%).

**TABLE 4:** Antimicrobials with ≥10 CPAs delivered in the study period and the proportion of recommended dosing adjustments.

Drug	No. of CPA	No. of patients	CPA/patient	Dosing adjustment		
				Increased	Decreased	None
Antibiotics						
Piperacillin-Tazobactam	36	9	4	6 (16.7%)	12 (33.3%)	18 (50.0%)
Meropenem	34	10	3.4	12 (35.3%)	2 (5.9%)	20 (58.8%)
Linezolid	21	6	3.5	3 (14.3%)	5 (23.8%)	13 (61.9%)
Teicoplanin	14	1	14	2 (14.3%)	0 (0.0%)	12 (85.7%)
Antifungals						
Isavuconazole	47	5	9.4	2 (4.3%)	0 (0.0%)	45 (95.7%)
Voriconazole	44	6	7.3	20 (45.5%)	11 (25.0%)	13 (29.5%)
Fluconazole	10	2	5	1 (10.0%)	0 (0.0%)	9 (90.0%)
Posaconazole	10	1	10	2 (20.0%)	0 (0.0%)	8 (80.0%)
Antivirals						
Ganciclovir	10	1	10	3 (30.0%)	1 (10.0%)	6 (60.0%)

CPA, clinical pharmacology advice.

**FIGURE 3:**
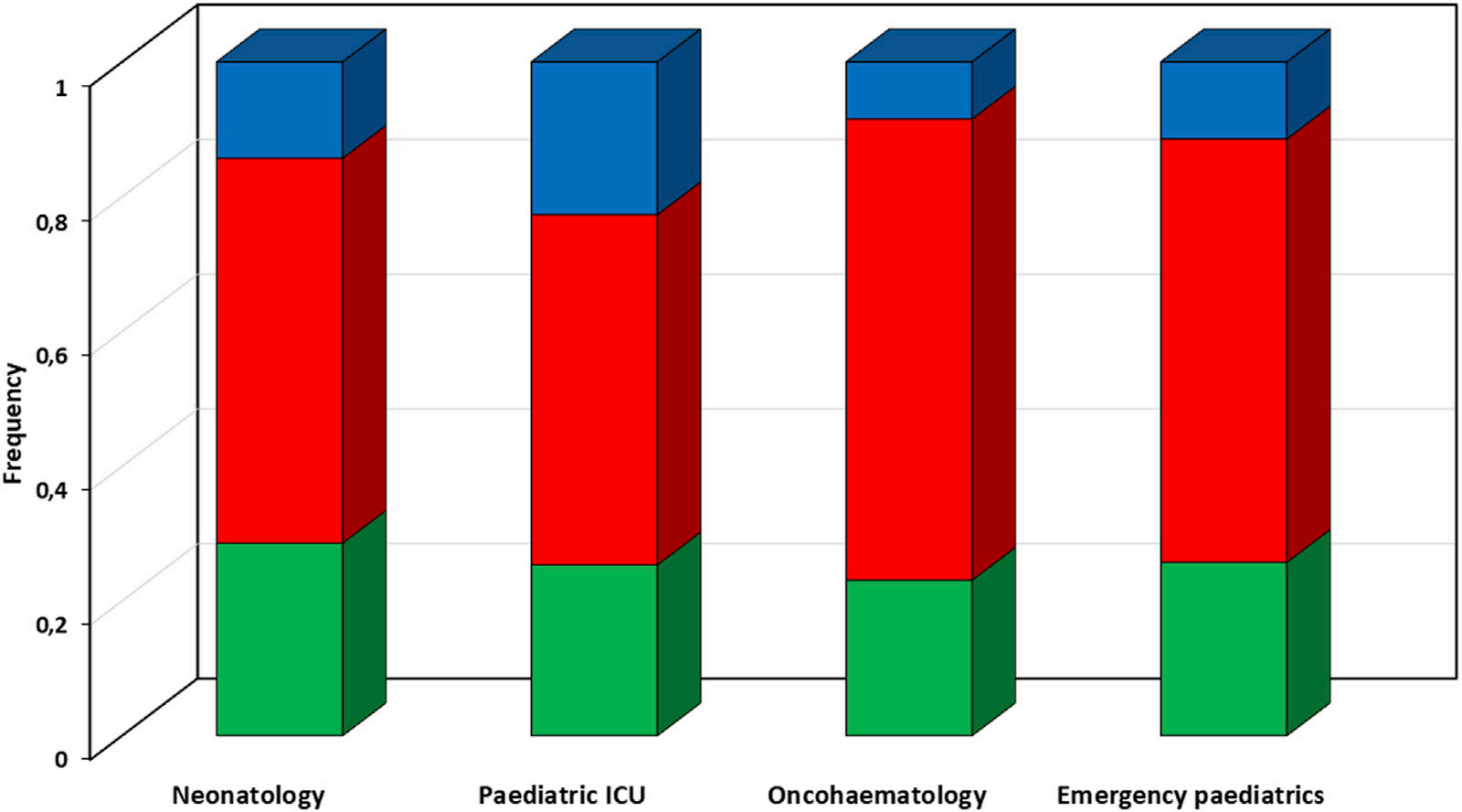
Proportion of types of recommended dosing adjustments according to different paediatric settings. Red box, suggested dosing increase; green box, suggested dosing confirm; blue box, suggested dosing reduction. ICU, intensive care unit.

Overall, the CPAs recommended dosing adjustments in 37.7% of cases, and suggested increases in 24.3% and decreases in the other 13.4% (Figure 3). Most of the dosing adjustments were needed in the paediatric ICU patients (48%), with increases and decreases almost equally distributed.

In regard to each single antimicrobial agent, dose adjustments were recommended in 70.5 and 50.0% of CPAs concerning voriconazole and piperacillin-tazobactam, respectively. Dose increases concerned mainly voriconazole (20 cases; 45.5%) and meropenem (12 cases; 35.3%), whereas dose reductions were needed mainly for piperacillin-tazobactam (12 cases; 33.3%) and voriconazole (11 cases; 25.0%). Conversely, dose adjustments were considered unnecessary in most of the CPAs delivered for isavuconazole (95.7%), fluconazole (90.0%), teicoplanin (85.7%), and posaconazole (80.0%).

**FIGURE 4:**
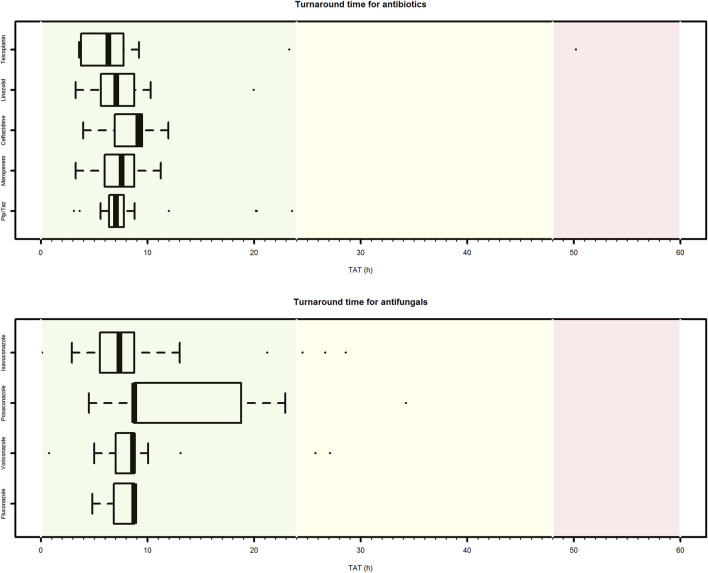
Box plot of turnaround time of clinical pharmacological advices (CPAs) for antimicrobial dosing regimen individualization in paediatric settings. Only antibiotic or antifungal agents for which at least ten CPAs were delivered are showed. Pip/Taz, piperacillin/tazobactam.

Median CPA turnaround time (TAT) was 7.5 h (IQR 6.1–8.8 h). No significant differences in median TAT was observed among the different paediatric setting (ranging from 6.4 h for neonatology to 8.2 h for paediatric onco-haematology/transplant unit) (Figure 4). Overall, CAP TAT was optimal in 91.5% of cases, and quasi-optimal in 96.0% of cases. Among the antimicrobials accounting for most of the delivered CPAs, the median TAT ranged between 6.4 h (IQR 4.1–7.5 h) for teicoplanin and 8.8 h (IQR 8.7–16.3 h) for posaconazole.

## Discussion

Our study provides a proof of concept of the helpful role that the CPAs based on TDM may have in optimizing antimicrobial exposure on real time in different populations and setting of hospitalized paediatric patient.

Both the indicators of performance of the CPAs may support the clinical utility of this approach. Dosing adjustments were needed in more than one third of cases, and the TAT of the CPAs was optimal in the vast majority of cases. This allowed clinicians to promptly implement recommended antimicrobial dosing adjustments in different challenging scenarios, and involved agents characterized by wide inter- and intraindividual variability according to specific pharmacokinetic-pharmacogenetic issues (e.g., voriconazole) (Bartelink et al., 2013) or underlying conditions (e.g., beta-lactams in critically ill children) (Marsot, 2018; Dhont et al., 2020), as found in our analysis. The prompt dosing adaptation may minimize either the risk of antimicrobial underexposure potentially associated with therapeutic failure, or those of overexposure potentially associated with toxicity.

In the last 10 years, TDM emerged as a valuable tool for assisting clinicians in optimizing antimicrobial dosing in different paediatric settings (Jager et al., 2016; Pauwels and Allegaert, 2016; John et al., 2019; De Rose et al., 2020; Hartman et al., 2020). The real added value of the CPA consists in the possibility of personalizing drug exposure on real-time in each single patient according to the antimicrobial susceptibility of the clinical isolate, the site of infection, the pathophysiological underlying conditions, and/or the drug-drug interactions of cotreatments. The innovative feature of this approach represents a paradigm shift in the TDM era. It is based on a multidisciplinary taskforce that may optimally handle complex hospitalized paediatric patients affected by severe infections, similarly to what just retrieved in other challenging scenarios (Viale et al., 2017; Gatti et al., 2019).

Antimicrobial use is quite frequent among hospitalized children, possibly exceeding 50% of cases in some paediatric settings (van Houten et al., 1998). Unfortunately, for most antimicrobials well-defined paediatric posology based on specific pharmacokinetic and/or pharmacokinetic/pharmacodynamic studies carried out in this patient population are currently lacking (Korth-Bradley, 2018) (Kearns et al., 2003; Ferro, 2015; De Rose et al., 2020). Consequently, our approach may represent the best way for dealing with the issue of appropriate treatment of infections in the paediatric setting. In this regard, we are confident that the implementation of TDM-guided CPAs for personalizing antimicrobial treatment could be of benefit especially in four challenging paediatric scenarios of infection.

### Sepsis and Septic Shock in Critically Ill Paediatric Patients

Sepsis and septic shock represent a major cause of ICU admission and mortality among critically ill paediatric patients (Garcia et al., 2020). The American College of Critical Medicine/Pediatric Advanced Life Support protocol recommends the administration of antibiotic therapy within the first hours of sepsis diagnosis (Davis et al., 2017). Although the adherence to this approach has been associated with improved patient care quality and reduced mortality (Weiss et al., 2014; Balamuth et al., 2016), the choice of appropriate antimicrobial dosing may result extremely challenging in this scenario. Consequently, the failure in achieving optimal PK/PD target may lead to an increased risk of antimicrobial inefficacy or toxicity.

Pathophysiological alterations in volume of distribution, plasma protein binding, and drug clearance, coupled with dynamic age-associated physiological variations in glomerular filtration rate and/or in drug metabolizing enzymes, may lead to non-attainment of the PK/PD targets of antimicrobials in the critically ill paediatric patients (Kearns et al., 2003; Marsot, 2018; Dhont et al., 2020; Hartman et al., 2020).

Our findings showing that dosing adjustments were needed in almost 50% of the CPAs delivered for ICU paediatric patients support this hypothesis. It has been shown that the prevalence of augmented renal clearance among critically ill children may be higher than 25% (Dhont et al., 2020). This means that the need for personalized TDM-guided CPAs could be remarkable especially for antimicrobials that are eliminated by the renal route, like beta-lactams, for which dosage increases are frequently required in this setting.

### Neonatal Sepsis

Neonatal sepsis is a systemic condition of bacterial, viral, or fungal etiology associated with haemodynamic changes and other clinical manifestations that may result in remarkable morbidity and mortality (Shane et al., 2017). Different causative agents may be identified in early-compared to late-onset sepsis, leading to different therapeutic strategies (Shane et al., 2017). Empirical therapy of early-onset neonatal sepsis is based on the combination of ampicillin plus an aminoglycoside, whereas that of late-onset neonatal sepsis is based on the combination of vancomycin plus an aminoglycoside (Shane et al., 2017). Additionally, linezolid could represent a valuable alternative in neonatal ICUs with high prevalence of vancomycin-resistant Enterococci (Subramanya et al., 2019).

Antimicrobial TDM may be very helpful in this challenging scenario, considering that in the newborns the relationship between drug dose and exposure is quite unpredictable, especially in the pre-terms, due to the abrupt developmental physiological changes that occur in this age period (Pauwels and Allegaert, 2016; De Rose et al., 2020). In this regard, several evidences support the potential role of TDM in the management of neonatal sepsis (Hoff et al., 2009; Touw et al., 2009; Sicard et al., 2015; Sosnin et al., 2019; Tauzin et al., 2019).

In our study CPAs in neonatal septic patients were performed only in seven cases, but it is worth noting that dosing adjustments were need in almost half of these. This may support the need for implementing personalized TDM-guided CPAs in this challenging scenario. Indeed, the low number of CPAs delivered in this setting is justified strictly by ethical and clinical restrictions. Venipuncture is distressing and painful in this fragile population (although in unstable neonates the umbilical vein is commonly catheterized), and extensive sampling should be avoided because of the risk of iatrogenic anemia possibly resulting in requirement for blood transfusions (De Rose et al., 2020). We are now dealing with this issue by implementing a new rapid mass spectrometry method based on capillary microsampling (50 µL) that will make feasible to quantify 14 different antibiotics in each microsample, as showed previously by Bacco et al. (Barco et al., 2020). This would greatly contribute in increasing the feasibility of TDM-based CPAs in the neonatal setting.

### Antifungal Prophylaxis or Treatment With Azoles in Onco-haematological Paediatric Patients

Invasive fungal infections represent a major cause of morbidity and mortality in children who are affected by onco-haematological malignancies or who need allogeneic haemopoietic stem-cell transplantation (Groll and Tragiannidis, 2010; Dvorak et al., 2012; Pana et al., 2017; Arad-Cohen et al., 2020; Groll et al., 2021). The breakdown in natural barriers due to mucositis and the need for indwelling catheters coupled with immunosuppression caused by underlying onco-haematological disease and/or myelosuppressive chemotherapy may represent major determinants of the increased risk of invasive fungal infections in this scenario (Dvorak et al., 2012).

*Aspergillus spp* are responsible for a large proportion of invasive fungal infections, followed by *Candida* species. Triazoles represent the most important class of antifungal agents for prophylaxis and treatment of invasive fungal infections (Groll and Tragiannidis, 2010). Unfortunately, the use of azoles is challenged by several pharmacokinetic and pharmacogenetic issues in the paediatric scenario (Bury et al., 2021). Large interindividual and intraindividual variability in azole exposure is frequently present among oncohaematologic paediatric patients, especially for voriconazole. Additionally, the remarkable risk of clinically relevant drug-drug interactions with immunosuppressants and/or chemotherapeutic agents may make the achievement of therapeutic targets of exposure even more difficult. TDM-guided approach has just been considered to play a relevant role in the optimization of antifungal prophylaxis/treatment among onco-haematological paediatric patients (Groll et al., 2017; Allegra et al., 2018; Lempers et al., 2019).

Our data support this, as in almost 30% of the CPAs delivered in onco-haematological paediatric dosing adjustments of voriconazole were needed. This approach could minimize the risk of antifungal underexposure that may cause breakthrough invasive fungal infections, and/or that of overexposure, possibly causing toxicity (e.g., hepatotoxicity), in relation to drug-drug interactions, pharmacogenetic issues, or disease-related organ failure (Kyriakidis et al., 2017; Bury et al., 2021).

### Community-Acquired Pneumonia (CAP) in Hospitalized Children

CAP is a common and potentially severe infection, with high incidence and relevant morbidity and mortality among children (McIntosh, 2002; Gupta et al., 2018), and is one of the major causes of admission to the emergency paediatric wards (Nascimento-Carvalho, 2020). Although respiratory viruses are the major causative pathogens in children under 5 years, also bacteria may play a relevant role. *Streptococcus pneumoniae*, *Haemophilus influenzae*, and *Mycoplasma pneumoniae* represent the most frequent bacterial pathogens (Gupta et al., 2018; Nascimento-Carvalho and Nascimento-Carvalho, 2019; Nascimento-Carvalho, 2020). Ampicillin represents the first-line antibiotic therapy in hospitalized children with CAP. Vancomycin or linezolid should be considered in presence of CA-MRSA (Gupta et al., 2018; Nascimento-Carvalho, 2020).

TDM could be a valuable tool in optimizing antibiotic therapy in paediatric patients with severe CAP admitted in the emergency ward, as suggested by the fact that in our study approximately 20% of CPAs were performed in this setting. Both ampicillin and vancomycin are hydrophilic antimicrobials whose pharmacokinetic variability could be considered high and unpredictable in this scenario. In this regard, antibiotic dosing adjustments were recommended in more than 60% of cases, even if we recognize that the total number of delivered CPAs was quite low. These findings may justify the need for implementing personalized TDM-guided CPAs in paediatric patients affected by severe pneumonia, similarly to that has been suggested for adults (Pea and Viale, 2006).

### Conclusion

In conclusion, our study provides a proof of concept of the helpful role that CPAs based on real-time TDM may have in optimizing antimicrobial exposure in different challenging paediatric scenarios. Although we recognize that the short duration of the study period and the limited sample size may be potential limitations, the non-negligible proportion of recommended dosing adjustments coupled with the optimal performance of TAT support the feasibility and usefulness of this approach. The forthcoming implementation of innovative methods based on capillary microsampling will make sample collection even more feasible in the paediatric setting, and this may hopefully lead to a relevant growth in the use of personalized TDM-guided CPAs for optimizing antimicrobial treatment.

## Data Availability

The raw data supporting the conclusions of this article will be made available by the authors, without undue reservation.
